# Mitochondrial Quality Control in Cardiac Diseases

**DOI:** 10.3389/fphys.2016.00479

**Published:** 2016-10-21

**Authors:** Juliane C. Campos, Luiz H. M. Bozi, Luiz R. G. Bechara, Vanessa M. Lima, Julio C. B. Ferreira

**Affiliations:** Department of Anatomy, Institute of Biomedical Sciences, University of Sao PauloSao Paulo, Brazil

**Keywords:** bioenergetics, oxidative stress, mitochondrial unfolded protein response, fusion-fission balance, mitophagy, aldehyde dehydrogenase 2, 4-hydroxynonenal

## Abstract

Disruption of mitochondrial homeostasis is a hallmark of cardiac diseases. Therefore, maintenance of mitochondrial integrity through different surveillance mechanisms is critical for cardiomyocyte survival. In this review, we discuss the most recent findings on the central role of mitochondrial quality control processes including regulation of mitochondrial redox balance, aldehyde metabolism, proteostasis, dynamics, and clearance in cardiac diseases, highlighting their potential as therapeutic targets.

## Introduction

Cardiac disease remains the leading cause of morbidity and mortality worldwide (Bayeva et al., 2013). Despite the fact that the mechanisms underlying the pathophysiology of heart disease are multiple and complex, recent research provides evidence that loss of mitochondrial homeostasis is a hallmark of cardiomyocyte dysfunction and death (Disatnik et al., 2013; Yogalingam et al., 2013).

Mitochondria are critical organelles for the maintenance of cardiac physiology since cardiomyocytes have a high demand for ATP synthesis and increased oxygen uptake rate. These mitochondrial features also make them the major source of reactive oxygen species (ROS) under pathological circumstances (Figueira et al., 2013). Indeed, disruption of mitochondrial electrochemical and redox properties is tightly associated with the onset and progression of cardiac pathophysiology (Palaniyandi et al., 2010; Gomes et al., 2015). Despite of their main role in ensuring that cardiac energy demands are met by energy supply, mitochondria have been lately highlighted as strategic intracellular nodes/transducers where signals converge. Mitochondria also serve as multi effector players in a wide range of intracellular signaling pathways that ultimately regulates nuclear gene expression, ions homeostasis, and apoptosis (Campos et al., 2013).

Considering the pivotal role of mitochondria in regulating intracellular homeostasis, eukaryotic cells have developed several mechanisms of surveillance capable of maintaining mitochondrial integrity and functionality upon stress (Baker et al., 2011; Kotiadis et al., 2014). These quality control machineries can be divided into three levels of surveillance. The first level of defense involves a multilayer network of detoxifying systems capable of fighting oxygen- and aldehyde-mediated mitochondrial toxicity. The second line of defense relies to mitochondrial proteases and chaperones responsible for the maintenance of mitochondrial proteostasis. The third level of defense involves the control of mitochondrial morphology and number through two interconnected processes: mitochondrial dynamics (fusion and fission) and mitophagy (mitochondrial clearance). These three levels of defense are well-conserved quality control mechanisms capable of regulating mitochondrial redox state, content, size and number due to changes in mitochondrial bioenergetics, biochemical, or electrochemical properties (Twig et al., 2008).

In this review, we will approach the aforementioned mechanisms of mitochondrial surveillance and defense in health and cardiac diseases; highlighting their critical role in the maintenance of cardiac homeostasis.

## Mitochondrial detoxifying systems (first level of defense)

Detoxifying systems are critical mitochondrial quality control mechanisms that provide protection from biogenic and xenogeneic molecules such as ROS and aldehydes.

The term oxidative stress describes conditions that result from the imbalance between free radical generation and its detoxification. Aerobic organisms have developed a series of antioxidant defenses, including antioxidant compounds and enzymes that directly react with and neutralize oxidizing agents; therefore minimizing the oxidative stress-induced cellular damage (Kornfeld et al., 2015).

Mitochondria are the main source of cellular ROS as well as have the highest antioxidant capacity (Figueira et al., 2013). Accumulation of mitochondrial ROS is critical to cardiac ischemia-reperfusion injury and heart failure (Campos et al., 2012, 2013; Chouchani et al., 2014). The use of experimental animal models has contributed to the understanding of the role of antioxidant system as a first line of defense against the pathogenesis of cardiac diseases. SOD deficiency has been linked to excessive ROS accumulation and cardiac mitochondrial dysfunction, thus contributing to the establishment of pathological ventricular hypertrophy and heart failure in mice (Strassburger et al., 2005; Morten et al., 2006; Lu et al., 2008). Moreover, cardiac overexpression of either MnSOD or Cu-ZnSOD isoforms attenuates cardiac ischemia-reperfusion injury and heart failure in mice (Chen et al., 1998; Miller et al., 2010).

In general, enhanced activity of enzymatic antioxidants (i.e., catalase, glutathione peroxidase, thioredoxin, and peroxiredoxin) or increased levels of non-enzymatic antioxidants (i.e., glutathione, N-acetylcysteine, ubiquinol, α-tocopherol, ascorbic acid, and lipoic acid) reduce cardiac oxidative stress in rodents by maintaining ROS at nanomolar levels (Goszcz et al., 2015). However, these findings have not been reproduced in randomized controlled trial (Kritharides and Stocker, 2002; Ye et al., 2013). One possible explanation for the failure in clinical trials relies to the inability of these antioxidants to properly counteract mitochondrial oxidative stress. Recent studies have demonstrated that the development of antioxidants that are target to and selectively accumulates within mitochondria attenuate mitochondrial-specific oxidative damage (Subramanian et al., 2010; Oyewole and Birch-Machin, 2015; Ni et al., 2016). Finally, antioxidant in excess should have detrimental effects on intracellular redox signaling, a ROS-mediated process that positively regulates a vast array of signaling pathways (Ristow et al., 2009).

**Figure 1:**
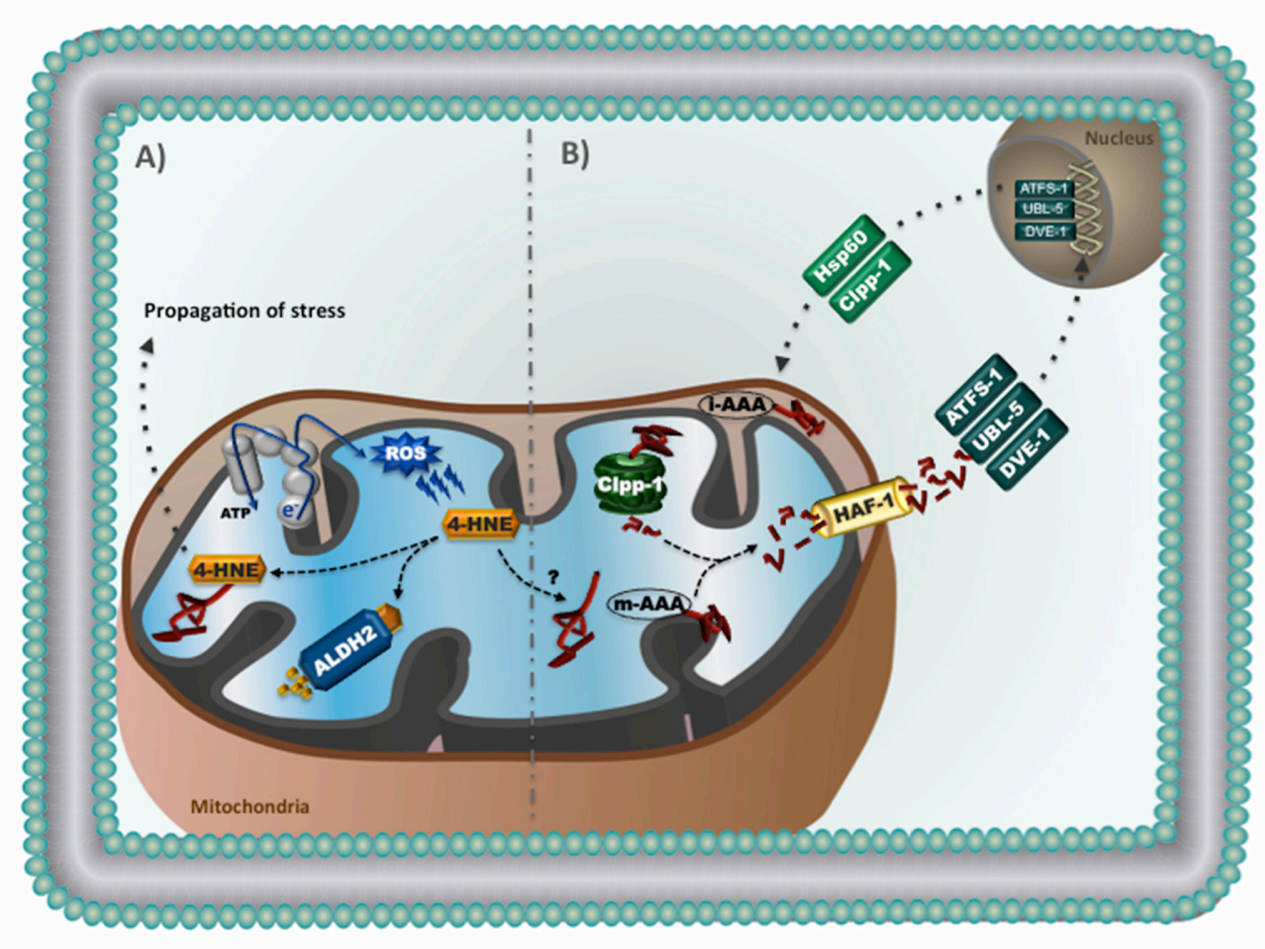
**(A)** Reactive aldehydes in mitochondria: excessive reactive oxygen species causes lipid peroxidation and consequent generation of 4-hydroxynonenal (4-HNE), a highly reactive aldehyde that readily forms protein adducts. Accumulation of 4-HNE adducts causes mitochondrial dysfunction and contributes to the propagation of stress related to cardiac pathophysiology. Aldehyde dehydrogenase 2 (ALDH2) is a mitochondrial enzyme responsible for the conversion of 4-HNE into inactive acids. **(B)** Mitochondrial unfolded protein response (UPR^mt^): Accumulation of mitochondrial unfolded proteins results in increased proteolysis and release of mitochondrial-generated peptides to the cytosol. It is suggested that these peptides activate cytosolic transcription factors (i.e., ATFS-1, UBL-5, and DVE-1). Once inside the nucleus, these transcription factors promote upregulation of genes involved in mitochondrial proteostasis (i.e., chaperones and proteases), thus relieving stress and re-establishing mitochondrial homeostasis. The UPR^mt^ has been characterized in the nematode *Caenorhabditis elegans*.

Recent findings revealed that not only elevated ROS are critical to cardiac diseases, but accumulation of endogenous toxic aldehydes also plays detrimental role in the cardiovascular system (Chen et al., 2010; Chen and Zweier, 2014). 4-hydroxynonenal (4-HNE), a major end-product of peroxidative degradation of mitochondrial phospholipids, is a highly reactive aldehyde that readily forms protein adducts via the Michael addition (Roede and Jones, 2010). Accumulation of 4-HNE causes cardiac mitochondrial dysfunction in a dose-dependent manner and contributes to cardiac pathophysiology in rodents (Campos et al., 2012; Gomes et al., 2014, 2015). Excessive 4-HNE adducts formation has also been reported in human failing hearts (Nakamura et al., 2002; Ferreira et al., 2012) and was associated with impaired cardiac bioenergetics, contractility properties and proteostasis (Campos et al., 2012; Ferreira et al., 2012). Therefore, accumulation of 4-HNE appears to be a hallmark of cardiac diseases (Figure 1A).

Aldehyde dehydrogenase 2 (ALDH2), a member of the aldehyde dehydrogenase family (Marchitti et al., 2008; Vasiliou et al., 2013), is a mitochondrial tetrameric enzyme responsible for the conversion of toxic aldehydes such as 4-HNE into inactive acids (Perez-Miller et al., 2010; Sobreira et al., 2011; Ferreira and Mochly-Rosen, 2012; Josan et al., 2013). ALDH2 has emerged as key enzyme in the protection of cardiac myocytes and neurons against aldehydic overload (Sun et al., 2011; Ferreira and Mochly-Rosen, 2012; Zambelli et al., 2014). Pharmacological ALDH2 activation using a small molecule (Alda-1) protects the heart against ischemia-reperfusion injury (Chen et al., 2008, 2014; Gross et al., 2015). We have recently reported that reducing cardiac aldehydic load via selective activation of mitochondrial ALDH2 is sufficient to improve ventricular function in heart failure through a better cardiac mitochondrial bioenergetics and reduced ROS generation (Gomes et al., 2014, 2015). These findings point out ALDH2 as a critical player in the maintenance of mitochondrial quality control, and highlight the potential therapeutic value of ALDH2 activators in counteracting the detrimental role of aldehydes in cardiac disease (Figure 1A).

## Mitochondrial proteostasis surveillance (second level of defense)

The endosymbiotic theory proposes that mitochondria (a former free-living proteobacterium) were engulfed by a precursor eukaryotic cell about 1.5 billion years ago (Gray et al., 1999). During the evolutionary process most of the mitochondrial genome was transferred to nuclear genome. Approximately 1100 mitochondrial proteins are encoded by the nucleus, translated in the cytosol and imported to mitochondria (Pagliarini et al., 2008; Baker et al., 2011). These proteins work in synchrony with the 13 mitochondrial-encoded proteins (all subunits of the electron transport chain) to maintain ATP production (Jovaisaite and Auwerx, 2015). Changes in the stoichiometric balance between nuclear- and mitochondrial-encoded proteins due to improper mitochondrial protein import and folding, as well as mutations in mitochondrial DNA, affect organelle proteostasis, which is sufficient to disrupt the integrity and functionality of mitochondria. Therefore, a network of mitochondrial chaperones and proteases are crucial for maintenance of organelle protein homeostasis.

Mitochondrial chaperones and chaperonins including mtHsp70, Hsp60, and Hsp10 are part of mitochondrial quality control system. These proteins assist mitochondrial protein import machinery, protein folding, electron transport chain assembly, and prevention of aggregation of unfolded proteins (Baker et al., 2011; Pellegrino et al., 2013). Loss of function of mitochondrial chaperones has been implicated in several pathological processes including cardiovascular diseases (MacKenzie and Payne, 2007). Elevated Hsp60 protein levels have been reported in human failing hearts, which might suggest a compensatory response due to disrupted mitochondrial proteostasis (Knowlton et al., 1998). Indeed, combined or individual overexpression of Hsp60 and Hsp10 protects mitochondrial function and prevents cardiomyocyte death induced by either hypoxia-reoxygenation injury or doxorubicin (Lin et al., 2001; Hollander et al., 2003; Shan et al., 2003).

Another critical component of mitochondrial quality control relies to proteolytic systems located at different mitochondrial compartments. Mitochondrial proteases are required to degrade misfolded, damaged, and unfolded proteins no longer capable of being refolded by chaperones. Lon and ClpXP are typical proteases located in the matrix. Lon degrades denatured and oxidized proteins while the substrates of ClpXP still have to be identified. Another class of proteases is located at mitochondrial inner membrane. These AAA metalloproteases (ATPases associated with a number of cellular activities) expose their catalytic sites either to matrix (m-AAA protease) or to intermembrane space (i-AAA protease) and are involved in processing and degradation of proteins (Griparic et al., 2007; Song et al., 2007; Bonn et al., 2011; Gerdes et al., 2012). Both i-AAA and m-AAA proteases mainly degrade unassembled mitochondrial intermembrane proteins (Baker et al., 2011). Disruption of i-AAA and m-AAA proteases affects mitochondria morphology, proteostasis, and bioenergetics (Nolden et al., 2005; Griparic et al., 2007). Recently, Wai et al. demonstrated that cardiac-specific ablation of the m-AAA protease YME1L alters mitochondrial morphology and metabolism and causes heart failure in mice (Wai et al., 2015).

Another important mitochondrial quality control mechanism is characterized by an inter-organelle communication between mitochondria and nucleus (known as mitochondrial retrograde signaling), which regulates mitochondrial proteostasis by upregulating nuclear-encoded mitochondrial genes (Figure 1B; Haynes et al., 2013). This process has been characterized and well-explored in the nematode *Caenorhabditis elegans*, where disruption of mitochondrial unfolded protein response (UPR^mt^) perturbs mitochondrial function and negatively affects longevity (Haynes et al., 2010). Briefly, loss of mitochondrial proteostasis triggers UPR^mt^, which may results in accumulation of mitochondrial-derived peptides in the cytosol and translocation of the transcriptional factor ATFS-1 to the nucleus. In association with others transcriptional factors (UBL-5 and DVE-1), ATFS-1 promotes upregulation of genes involved in mitochondrial proteostasis (i.e., chaperones and proteases), glycolytic metabolism, detoxification, and innate immune response, thus relieving stress and re-establishing mitochondrial homeostasis (Benedetti et al., 2006; Jovaisaite et al., 2014; Nargund et al., 2015).

Fiorese et al. recently described how UPR^mt^ is regulated in mammals (Fiorese et al., 2016). They suggest that ATF5, a mammalian transcriptional factor, has a protective role during mitochondrial dysfunction by regulating UPR^mt^ signaling, similar to ATFS-1 in worms. The role of UPR^mt^ signaling in cardiovascular diseases is unknown. It is expected that UPR^mt^ signaling might be activated in failing hearts or during ischemia-reperfusion injury to compensate impaired mitochondrial proteostasis. In fact, UPR^mt^ activation protects *C. elegans* against ischemic injury (Durieux et al., 2011; Kaufman and Crowder, 2015).

## Mitochondrial dynamics and mitophagy (third level of defense)

**Figure 2:**
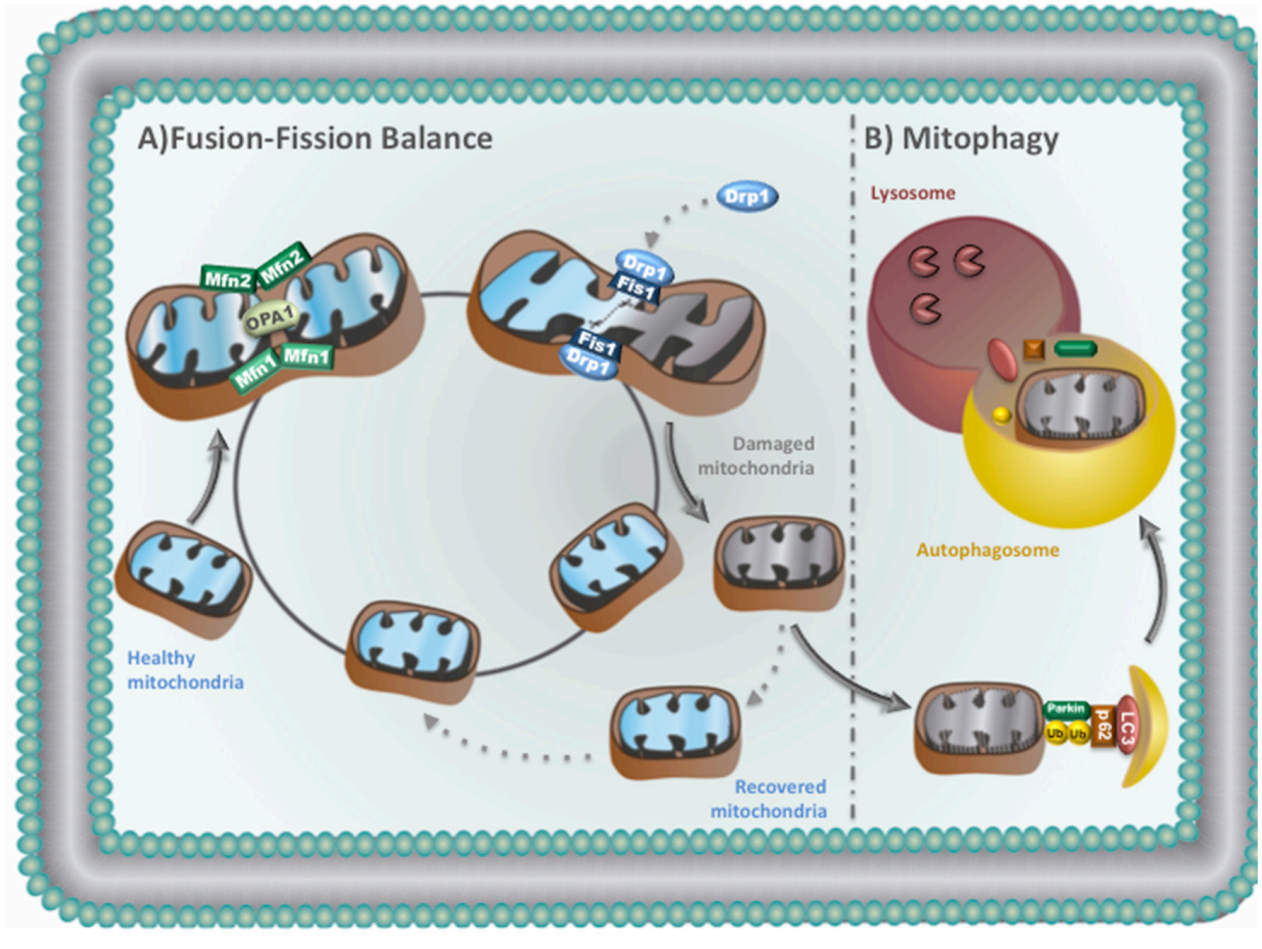
**(A)** Mitochondrial fusion-fission balance: Mitochondrial fission: Drp1 translocates to mitochondria and binds to Fis1 to promote fission. Daughter mitochondria can be recovered or removed. Recovered mitochondria re-enter in the life cycle to fuse with healthy mitochondria. Mitochondrial fusion: outer membrane fusion is regulated by Mfn1 and Mfn2, while OPA1 is responsible for inner mitochondrial membrane fusion. **(B)** Mitophagy: damaged mitochondria can be sequestered by autophagosomes. The autophagosomes then fuse with lysosomes to degrade sequestered mitochondria.

Mitochondria usually form a highly dynamic network that plays crucial role in cardiac bioenergetics. The maintenance of mitochondrial size, shape, number and location relies to well-conserved quality control mechanisms including mitochondrial fusion-fission machinery, and mitophagy (Figure 2; Liesa et al., 2009; Dorn and Kitsis, 2015; Shirihai et al., 2015). Disruption of these quality control processes result in accumulation of abnormal mitochondria, which is likely associated to cardiovascular diseases (Dorn, 2013; Andres et al., 2015).

### Fusion machinery

Mitochondrial fusion is important for the exchange of DNA, proteins and metabolites between two neighboring mitochondria, therefore helping the maintenance of oxidative phosphorylation, membrane potential and DNA replication/repair (Liesa et al., 2009). Mitochondrial fusion requires both the outer and the inner membranes of two mitochondria to fuse (Disatnik et al., 2015). Two members of the large GTPase dynamin family orchestrate the fusion of the outer mitochondrial membrane (mitofusins 1 and 2—Mfn1 and Mfn2) while OPA1 (optic atrophy factor 1) mediates inner membrane fusion (Figure 2). OPA1 is subjected to proteolytic processing where accumulation of short forms is accompanied by fragmentation of mitochondria network and loss of cristae structures (Griparic et al., 2007; van der Bliek et al., 2013). Despite of its role in regulating mitochondrial dynamics, Mfn2 also links mitochondria to the endoplasmic reticulum, a critical process for controlling calcium handling and cell death in cardiomyocytes (Dorn et al., 2015).

Mitochondrial fusion is critical for heart physiology. Combined Mfn1 and Mfn2 ablation of mouse hearts results in accumulation of fragmented mitochondria and leads to heart failure (Chen et al., 2011). Of interest, Hall et al. recently demonstrated that double Mfn1/Mfn2 knockout animals are protected from acute ischemia-reperfusion due to impaired mitochondria/sarcoplasmic reticulum tethering (Hall et al., 2016). These findings suggest that Mfn1 and Mfn2 may play either protective or detrimental role according to stress condition. Disruption of mitochondrial dynamics through deletion of OPA1 or imbalanced OPA1 processing also causes mitochondrial fragmentation, bioenergetics deficit and heart failure in mice (Chen et al., 2012; Wai et al., 2015). Overall, enzymes that control mitochondrial fusion are critical for the maintenance of bioenergetics, cellular viability, and cardiac function. Therefore, pharmacological and non-pharmacological interventions that modulate Mfn1, Mfn2 and OPA1 may have cardioprotective effects against acute and chronic pathological conditions.

### Fission machinery

Fission is critical for segregating dysfunctional mitochondria, where the impaired daughter organelle can be either recovered by fusing with healthy mitochondria or eliminated through autophagy (Gottlieb and Bernstein, 2016; Figure 2). Mitochondrial fission is triggered by dynamin-related protein 1 (Drp1). Upon activation, Drp1 translocates from the cytosol to the outer mitochondria membrane, binds to adaptor proteins and constrict both mitochondrial outer and inner membranes (Suzuki et al., 2005; Otera et al., 2010). Impaired mitochondrial fission due to a missense mutation in Dnm1 (Drp1) causes progressive energy deficient and dilated cardiomyopathy in rodents (Ashrafian et al., 2010).

Excessive mitochondrial fission has a negative impact on heart during ischemia-reperfusion injury (Disatnik et al., 2015). Inhibition of mitochondrial fission using a dominant-negative mutant form of Drp1 protects cultured cardiomyocytes against hypoxia-reoxygenation stress (Ong et al., 2010). More recently, we demonstrated that the use of a selective inhibitor of the fission machinery (p110 peptide; Qi et al., 2013), which inhibits the interaction of fission proteins Fis1/Drp1, decreases mitochondrial fission and improves bioenergetics in different models of ischemia-reperfusion injury, including primary cardiomyocytes, *ex vivo* heart model, and an *in vivo* myocardial infarction model (Disatnik et al., 2013). Of interest, a single dose of p110 peptide at reperfusion after transient coronary artery occlusion is sufficient to inhibit excessive mitochondrial fragmentation, increase mitochondrial oxygen consumption, and improve cardiac function in the long-term (Disatnik et al., 2013). Together, these studies provide evidence that blocking excessive mitochondrial fission protects the heart against acute ischemia-reperfusion injury in rodents. Therefore, therapies capable of reducing excessive mitochondrial fragmentation may become a valuable tool against myocardial infarction.

### Mitophagy

Accumulation of dysfunctional mitochondria is suggested to play a key role in cardiac pathophysiology. Therefore, turnover of mitochondria is necessary to maintain cardiac homeostasis. Impaired mitochondria can be selectively targeted and eliminated through a process termed mitochondrial autophagy (mitophagy; Figure 2B). Briefly, in healthy mitochondria PINK1 (PTEN-induced putative kinase 1) is targeted to the mitochondrial inner membrane where it is degraded. During stress, dissipation of mitochondrial membrane potential results in accumulation of PINK1 on the mitochondrial surface, which phosphorylates/activates the E3 ubiquitin ligase Parkin (Kane et al., 2014). Parkin ubiquitinates key proteins that can be degraded by the proteasome or recognized by autophagy adaptor proteins. These adaptor proteins are able to act as cargo receptors, which recruit them to autophagosomes (Lazarou et al., 2015). The autophagosomes then fuse with lysosomes to degrade sequestered mitochondria (Figure 2B; Youle and Narendra, 2011). This process is known as macroautophagy. Mitochondria can also be directly engulfed through membrane invagination by lysosome, a process called microautophagy (Yogalingam et al., 2013; Hwang et al., 2015). Both macroautophagy and microautophagy are critical to remove damaged mitochondria during cardiac stress (Disatnik et al., 2015).

Markers of autophagy are upregulated in cardiac diseases (Nakai et al., 2007; Zhu et al., 2007) However, accumulation of these proteins may reflect either increased or insufficient autophagy flux (Klionsky et al., 2016). Studies using genetically modified animals provide evidence that autophagy is critical for the maintenance of cardiac homeostasis (Kubli et al., 2013; Gong et al., 2015). Tissue-specific knockout of autophagy-related genes causes accumulation of disorganized mitochondria and oxidative stress (Tanaka et al., 2000; Wu et al., 2009) whereas overexpression of these genes ameliorates cardiomyopathy (Bhuiyan et al., 2013).

Disruption of mitophagy in Parkin-deficient hearts is sufficient to cause accumulation of dysfunctional mitochondria, oxidative stress, apoptosis, left ventricular dysfunction, and pathological cardiac hypertrophy (Gong et al., 2015). Moreover, defective mitophagy exacerbates cardiac damage induced by myocardial infarction (Narendra et al., 2008). These findings suggest that proper elimination of damaged mitochondria during stress conditions (i.e., myocardial infarction or heart failure) protect against oxidative stress and apoptosis; therefore contributing to the maintenance of cardiac physiology. Of interest, mitophagy markers are reduced in end-stage human heart failure (Billia et al., 2011). Saito et al. recently demonstrated that reduced autophagic vacuoles in cardiomyocytes are associated with poor heart failure prognosis in humans (Saito et al., 2016).

Finally, mitophagy seems to play a critical role during acute cardiac ischemia-reperfusion injury, a process characterized by accumulation of damaged mitochondria and severe oxidative stress. Pharmacological upregulation of autophagy prevents the onset of cell death following ischemia-reperfusion injury (Hamacher-Brady et al., 2006; Sala-Mercado et al., 2010; Yogalingam et al., 2013). However, future studies are required to test whether this cardioprotective effect occurs through enhanced clearance of damaged mitochondria. Of interest, depletion of either Parkin or p62/SQTSTM1 is sufficient to abolish the cardioprotective effects of both ischemic preconditioning and simvastatin treatment in rodents (Huang et al., 2011; Andres et al., 2014).

## Summary and perspectives

Mitochondria are essential organelles for the maintenance of myocardial homeostasis. They play critical role in bioenergetics, redox balance, ion homeostasis, and cell death. The importance of functional mitochondrial to the heart has been highlighted by the fact that situations that lead to mitochondrial dysfunction are often associated with cardiac diseases. Many of these diseases manifest later in life, where mitochondria seems to be less functional. Therefore, different levels of mechanisms of surveillance and quality control capable of detecting and fixing defects that affect mitochondrial performance are critical for the maintenance of long-lived cells with high energy demand such as cardiomyocytes. The central role of mitochondrial quality control in the health of myocardium has been recently reported. As described above, the machinery regulating mitochondrial quality control including mitochondrial redox balance, aldehyde metabolism, proteostasis, dynamics, and clearance are potential novel therapeutic targets for cardiac diseases. However, future research focusing on the critical molecular events involved in mitochondrial quality control is needed to develop better pharmacological interventions.

## Author contributions

JCC, LHMB, LRGB, and VL wrote the manuscript. JCBF designed, supervised, and wrote the manuscript.

### Conflict of interest statement

The authors declare that the research was conducted in the absence of any commercial or financial relationships that could be construed as a potential conflict of interest.
